# Increased levels of LAPTM4B, VEGF and survivin are correlated with tumor progression and poor prognosis in breast cancer patients

**DOI:** 10.18632/oncotarget.17176

**Published:** 2017-04-18

**Authors:** Sha Li, Lu Wang, Yue Meng, Yanli Chang, Jianjun Xu, Qingyun Zhang

**Affiliations:** ^1^ Department of Clinical Laboratory, Key Laboratory of Carcinogenesis and Translational Research, Ministry of Education, Peking University Cancer Hospital & Institute, Beijing 100142, China

**Keywords:** breast cancer, LAPTM4B, VEGF, survivin, prognosis

## Abstract

**Objective:**

This study explored the relationships among the expression of LAPTM4B, VEGF, and survivin and clinicopathological characteristics and prognosis in breast cancer patients.

**Methods:**

The expression of these three molecules in 110 stage I-III breast cancer patients with clinicopathological and follow-up data was detected via immunohistochemistry. Kaplan-Meier and Cox proportional hazard regression analyses were performed to assess the prognostic significance of these markers in breast cancer. Moreover, expression levels of these markers were evaluated in 5 breast cell lines via Western blot analysis.

**Results:**

LAPTM4B, VEGF, and survivin were over-expressed in breast cancer specimens and highly expressed in MDA-MB-231 cells. VEGF and nuclear survivin expression was significantly correlated with LAPTM4B expression, and high levels of all three were associated with a tumor size >2cm, TNM stage II+III and lymph node metastasis, which had worse impacts on overall survival and progression-free survival in breast cancer patients. A multivariate Cox analysis identified LAPTM4B over-expression as an independent prognostic marker in breast cancer.

**Conclusions:**

These findings suggest that LAPTM4B, VEGF, and nuclear survivin expression are significantly correlated in breast cancer, which may be predictive of prognosis as well as effective therapeutic targets for new anticancer therapies.

## INTRODUCTION

Breast cancer is one of the most common cancers among women, and epidemiological statistics show that the incidence of this disease and its associated mortality are increasing yearly [1]. The identification of key genes that determine progression and metastasis in breast cancer is urgently needed for early diagnoses and molecular targeted therapies.

Lysosome-associated protein transmembrane-4 beta (LAPTM4B), a novel oncogene that belongs to the mammalian 4-tetra-transmembrane spanning protein superfamily, was initially identified in human hepatocellular carcinoma [2, 3]. Previous studies showed that LAPTM4B-35 activity was elevated in various malignant tumors, which was associated with poor prognosis [4–6]. Moreover, it was indicated that LAPTM4B could increase the proliferation and metastasis of tumor cells, reduce apoptosis, and assist drug resistance, which involved in activated PI3K/AKT and Ras-MAPK signaling pathways [7].

Angiogenesis is an important feature of carcinogenesis, progression and metastasis in many human malignancies. Vascular endothelial growth factor (VEGF) is thought to be a major mediator of angiogenesis that promotes the proliferation of tumor cells and boosts invasion and metastasis via the activation of the PI3K/AKT pathway [8–10]. The vascular density of tumors, including breast cancer, has been proven to be closely correlated with prognosis [11].

Survivin is a 16.5-kDa intracellular protein that is a well-known member of the inhibitor of apoptosis protein family, and its expression is elevated in the majority of tumors [12]. It potentially facilitates cell cycle processes and cell division and reduces apoptotic indices, which is strongly related with poor prognosis in breast cancer [13, 14]. Transcription from the survivin gene locus gives rise to 5 splice variants [15]. The relation of the splicing variants with prognosis is currently unclear.

Interestingly, a previous study revealed that tissues with elevated expression of LAPTM4B had significantly more new capillary blood vessels than tissues with reduced expression in a mouse xenograft model of liver cancer, indicating that LAPTM4B over-expression might be significantly associated with increased angiogenic activity [16]. Recent studies have also shown that silencing LAPTM4B remarkably reduces the expression of VEGF in HeLa cells [17] and that increased LAPTM4B-35 combined with positive VEGF expression might serve as a new biological marker to predict outcomes in cervical carcinoma [18]. In addition, it has been proven that the upregulation of LAPTM4B-35 promotes the activation of AKT and Bad, which would maintain cell survival [16].

The correlations among LAPTM4B, VEGF, and survivin have not been investigated in breast cancer. Therefore, we performed a retrospective study including 110 breast cancer patients who underwent surgical resection, primarily exploring relationships among the expression of these three markers, clinical variables and survival.

## RESULTS

### Expression of LAPTM4B, VEGF, and survivin

**Figure 1:**
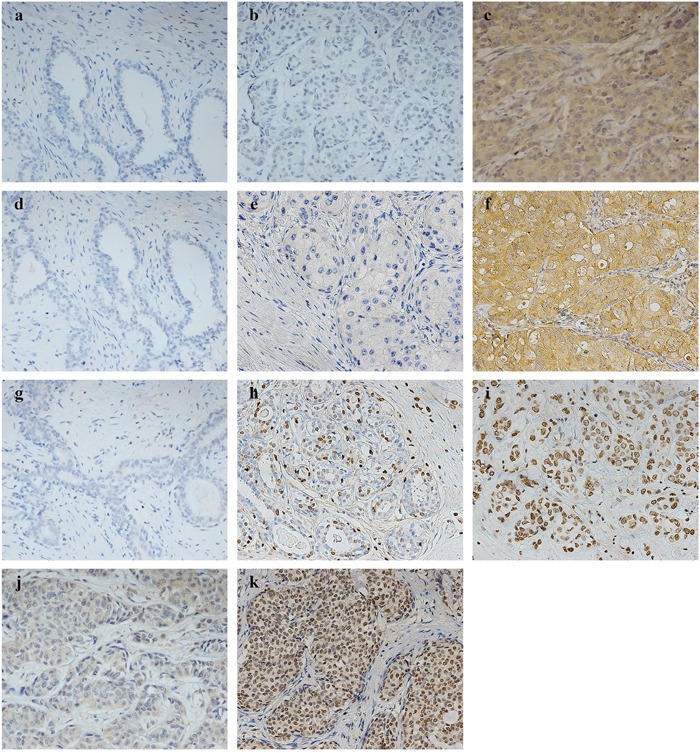
Representative pictures by immunohistochemistry for LAPTM4B, VEGF, and survivin in 110 breast cancer patients LAPTM4B expression: **(a)** low expression in benign breast tumor specimens, **(b)** low expression in breast cancer, **(c)** high expression in breast cancer. VEGF expression: **(d)** low expression in benign breast tumor specimens, **(e)** low expression in breast cancer, **(f)** high expression in breast cancer. Survivin expression: **(g)** low expression in benign breast tumor specimens, **(h)** low nuclear expression in breast cancer, **(i)** high nuclear expression in breast cancer, **(j)** only cytoplasmic expression in breast cancer, **(k)** both nuclear and cytoplasmic expression in breast cancer. (a–k, original magnification, ×200).

Statistical analyses showed that the expression of these three markers was significantly elevated in breast cancer specimens (P<0.05) (Table 1). As shown in Figure 1a-1k, LAPTM4B and VEGF protein staining were localized in the cytoplasm. Survivin staining differed among the cases: 63 cases (57.27%) showed expression only in the nucleus, 21 (19.09%) only in the cytoplasm, and 17 (15.45%) in both the nucleus and cytoplasm. Additionally, high VEGF and nuclear survivin protein expression were linearly correlated with that of LAPTM4B (P<0.001), as shown in Table 2.

**Table 1:** Expression of LAPTM4B, VEGF and survivin in breast tissue specimens

Groups	N	LAPTM4B expression		P^a^	VEGF expression		P^a^	Survivin expression		P^a^
		High expression	%		High expression	%		High expression	%	
Benign breast tumor	10	1	10	0.013*	1	10	0.007*	3	30	0.038*
Breast cancer	110	62	56.3		66	60.0		75	68.1	

^a^Calculated by χ2 test.

*P < 0.05.

**Table 2:** Correlations among LAPTM4B, VEGF and survivin in 110 breast cancer patients

	Total no.	LAPTM4B		P^a^
		Low expression	High expression	
VEGF				< 0.001*
Total no.	110	48	62	
Low expression	44	14	52	
High expression	66	34	10	
Nuclear survivin				< 0.001*
Total no.	89	35	54	
Low expression	27	12	50	
High expression	62	23	4	
Cytoplasmic survivin				0.103
Total no.	47	33	14	
Low expression	22	15	10	
High expression	25	18	4	

^a^Calculated by χ^2^ test.

*P < 0.001.

**Figure 2:**
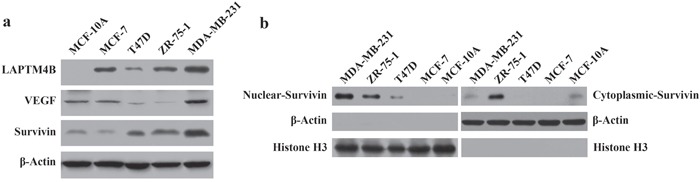
LAPTM4B, VEGF, nuclear and cytoplasmic survivin protein expression in breast cell lines by Western blot analysis Protein samples were obtained from 5 breast cell lines (MDA-MB-231, ZR-75-1, T47D, MCF-7 and MCF-10A). **(a)** β-Actin was used as an internal control for the total protein. High expression of LAPTM4B, VEGF and survivin was detected in MDA-MB-231 cells. **(b)** Histone H3 and β-Actin were used as an internal control for the nuclear and cytoplasmic protein samples, respectively. High levels of survivin were expressed in the nuclear protein of MDA-MB-231 cells.

Figure 2a shows that LAPTM4B, VEGF and survivin expression levels were increased in MDA-MB-231 cells, which were more highly metastatic than the other cell lines. Furthermore, we extracted the nuclear and cytoplasmic fractions in 5 breast cell lines and found that survivin protein expression was highest in the nuclear fractions of highly malignant MDA-MB-231 cells. For the cytoplasmic protein samples, survivin expression in MDA-MB-231 cells was lower than in the other cell lines (Figure 2b).

### Relationships between LAPTM4B, VEGF, and survivin protein expression and clinicopathological factors in breast cancer patients

As shown in Table 3, high levels of LAPTM4B, VEGF, and nuclear survivin were found in cases where factors related to tumor progression were present, such as tumor sizes >2 cm, TNM stage II+III and lymph node metastasis. Moreover, there was a significantly positive correlation between LAPTM4B and VEGF expression levels and the probability of tumor-associated venous thrombus (P=0.012 and P<0.001, respectively).

**Table 3:** Associations between the expression levels of LAPTM4B, VEGF and survivin and clinicopathological factors in 110 breast cancer patients

Characteristics	LAPTM4B expression	P^a^	VEGF expression	P^a^	Nuclear survivin expression	P^a^	Cytoplasmic survivin expression	P^a^
	High/Low		High/Low		High/Low		High/Low	
Patient No.	62/48		66/44		62/27		25/22	
Age (years)		0.290		0.936		0.424		0.344
≤55	21/21		25/17		22/12		8/10	
>55	41/27		41/27		40/15		17/12	
Menopausal status		0.178		0.507		0.279		0.351
Pre-menopausal	17/19		20/16		18/11		7/9	
Post-menopausal	45/29		46/28		44/16		18/13	
Tumor size (cm)		<0.001*		<0.001*		0.001*		0.355
≤2	8/24		11/21		13/15		6/8	
>2	54/24		55/23		49/12		19/14	
Histological type		0.607		0.350		0.873		0.280
IDC	55/41		56/40		52/23		22/22	
Others	7/7		10/4		10/4		3/0	
Histological grade		0.053		0.089		0.230		0.697
G1	5/10		6/9		8/7		2/2	
G2/G3	57/38		60/35		54/20		23/20	
TNM stage		<0.001*		<0.001*		0.001*		0.355
I	6/23		9/20		11/14		6/8	
II/III	56/25		57/24		51/13		19/14	
Lymph node metastasis		<0.001*		<0.001*		<0.001*		0.072
No	16/33		20/29		19/19		13/17	
Yes	46/15		46/15		43/8		12/5	
Tumor thrombus in vena		0.012*		<0.001*		0.607		0.058
No	38/40		38/40		45/21		14/18	
Yes	24/8		28/4		17/6		11/4	
ER status		0.121		0.234		0.077		0.137
Negative	15/6		15/6		12/2		8/3	
Positive	47/42		51/38		50/25		17/19	
PR status Negative	20/14	0.727	22/12	0.656	18/8	0.954	10/8	0.798
Positive	42/34		44/32		44/19		15/14	
Her-2 status		0.130		0.119		0.478		0.556
Negative	44/40		47/37		48/19		20/16	
Positive	18/8		19/7		14/8		5/6	
Recurrence		0.336		0.044*		0.090		0.855
No	47/40		48/39		45/24		21/19	
Yes	15/8		18/5		17/3		4/3	

^a^Calculated by χ^2^ test.

*P < 0.05.

### Univariate and multivariate survival analyses

**Figure 3:**
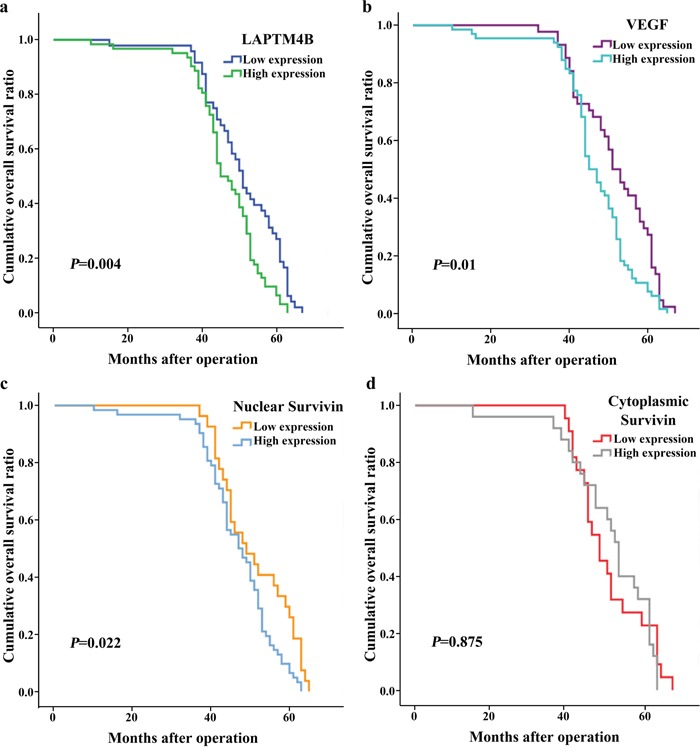
Kaplan-Meier curves for overall survival in 110 patients with breast cancer High expression of LAPTM4B **(a)**, VEGF **(b)** and nuclear survivin **(c)** was significantly associated with poor overall survival (P = 0.004, 0.01 and 0.022).

**Figure 4:**
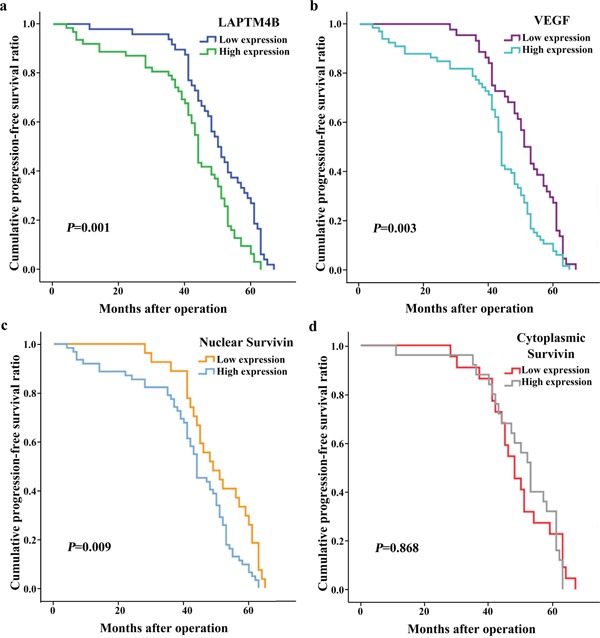
Kaplan-Meier curves for progression-free survival in 110 patients with breast cancer The progression-free survival was statistically shorter in groups with elevated expression of LAPTM4B **(a)**, VEGF **(b)** and nuclear survivin **(c)** (P = 0.001, 0.003 and 0.009). Expression of cytoplasmic survivin **(d)** was not related to the progression-free survival.

As shown by the survival analyses, death and recurrence occurred in 12 (10.91%) and 23 cases (20.91%), respectively. The Kaplan–Meier and log-rank tests showed that tumor sizes >2 cm and lymph node metastasis were correlated with poor overall survival (OS) and progression-free survival (PFS) in breast cancer (Table 4; Figures 3 and 4). A more advanced TNM stage was closely associated with a significantly worse PFS. The univariate model indicated that OS and PFS were significantly lower in cases with elevated LAPTM4B, VEGF, and nuclear survivin levels than in cases with lower levels of these molecules.

**Table 4:** Univariate Kaplan–Meier survival analysis of OS and PFS in 110 breast cancer patients

Variables	N	OS (months)		P^a^	PFS (months)		P^a^
		Mean ± SE	95 % CI		Mean ± SE	95 % CI	
Age (years)				0.437			0.366
≤55	42	50.000±1.561	46.941-53.059		48.000±3.121	41.882-54.118	
>55	68	46.000±2.257	41.577-50.423		44.000±1.805	40.463-47.537	
Menopausal status				0.186			0.183
Pre-menopausal	36	51.000±1.420	48.218-53.782		50.000±2.860	44.394-55.606	
Post-menopausal	74	46.000±2.121	41.842-50.158		44.000±1.885	40.306-47.694	
Tumor size (cm)				0.027*			0.009*
≤2	32	52.000±1.697	48.674-55.326		52.000±1.697	48.674-55.326	
>2	78	45.000±1.778	41.514-48.486		43.000±1.068	40.907-45.093	
Histological type				0.613			0.665
IDC	96	48.000±1.854	44.367-51.633		46.000±1.722	42.625-49.375	
Others	14	51.000±4.330	42.513-59.487		47.000±6.062	35.118-58.882	
Histological grade				0.390			0.333
G1	15	52.000±3.146	45.835-58.165		52.000±1.797	48.477-55.523	
G2/G3	95	48.000±1.914	44.248-51.752		44.000±1.499	41.063-46.937	
TNM stage				0.077			0.033*
I	29	52.000±2.596	46.911-57.089		52.000±2.596	46.911-57.089	
II/III	81	47.000±2.205	42.678-51.322		44.000±0.976	42.087-45.913	
Lymph node metastasis				0.021*			0.010*
No	49	51.000±1.916	47.244-54.756		51.000±2.000	47.080-54.920	
Yes	61	45.000±1.760	41.550-48.450		44.000±1.439	41.179-46.821	
Tumor thrombus in vena				0.366			0.315
No	78	49.000±1.612	45.840-52.160		48.000±1.881	44.313-51.687	
Yes	32	47.000±3.339	40.456-53.544		44.000±2.782	38.546-49.454	
ER status				0.201			0.190
Negative	21	45.000±1.831	41.411-48.589		43.000±1.144	40.757-45.243	
Positive	89	50.000±1.347	47.360-52.640		48.000±1.886	44.304-51.696	
PR status				0.841			0.931
Negative	34	47.000±2.328	42.436-51.564		43.000±1.458	40.143-45.857	
Positive	76	50.000±1.337	47.379-52.621		48.000±1.743	44.584-51.416	
Her-2 status				0.982			0.669
Negative	84	49.000±1.309	46.434-51.566		48.000±1.666	44.735-51.265	
Positive	26	45.000±2.550	40.003-49.997		44.000±3.389	37.357-50.643	
LAPTM4B				0.004*			0.001*
Low	48	51.000±1.954	41.170-54.830		50.000±1.873	46.329-53.671	
High	62	45.000±1.687	41.693-48.307		44.000±0.781	42.470-45.530	
VEGF				0.010*			0.003*
Low	44	51.000±1.873	47.329-54.671		51.000±1.561	47.941-54.059	
High	66	45.000±1.195	42.658-47.342		44.000±0.446	43.126-44.874	
Nuclear survivin				0.022*			0.009*
Low	27	48.000±3.674	40.799-55.201		48.000±3.674	40.799-55.201	
High	62	47.000±2.789	41.534-52.466		44.000±0.970	42.099-45.901	
Cytoplasmic survivin				0.875			0.868
Low	22	48.000±2.152	43.782-52.218		48.000±2.152	43.782-52.218	
High	25	53.000±1.208	50.633-55.367		53.000±3.019	47.083-58.917	

OS, overall survival; PFS, progression-free survival; CI, confidence interval.

^a^Log-rank test.

*P < 0.05.

The multivariate analysis showed that high levels of LAPTM4B were an independent prognostic marker for both OS and PFS in breast cancer (Table 5; P=0.007 and P=0.002, respectively).

**Table 5:** Multivariate Cox regression analysis of various predictive factors for OS and PFS in 110 breast cancer patients

Variables	OS (months)			PFS (months)		
	RR	95 % CI	P^a^	RR	95 % CI	P^a^
Tumor size (cm)	N/A	N/A	N/A	N/A	N/A	N/A
TNM stage	N/A	N/A	N/A	N/A	N/A	N/A
Lymph node metastasis	N/A	N/A	N/A	N/A	N/A	N/A
LAPTM4B	1.730	1.163-2.574	0.007*	1.839	1.239-2.730	0.002*
VEGF	N/A	N/A	N/A	N/A	N/A	N/A
Nuclear survivin	N/A	N/A	N/A	N/A	N/A	N/A
Cytoplasmic survivin	N/A	N/A	N/A	N/A	N/A	N/A

OS, overall survival; PFS, progression-free survival; RR, relative risk; CI, confidence interval.

^a^ Cox regression test.

*P < 0.05.

## DISCUSSION

Despite the fact that an increasing number of genes have been discovered and various targeted therapies have also been developed in recent years, the survival rate for breast cancer is not satisfactory [19]. Thus, it is of great importance to identify biomarkers that are effective in helping to improve the prognosis in breast cancer.

A number of studies have shown that the proliferation of cells overexpressing LAPTM4B is closely correlated with tumor progression and metastasis [20, 21]. In our study, increased LAPTM4B expression was strongly associated with prognosis-related features, including tumor size, TNM stage and lymph node metastasis. Further analysis revealed that breast cancer patients with high levels of LAPTM4B protein expression had worse OS and PFS rates.

Our study found that the relationship between VEGF and nuclear survivin expression levels and the expression of LAPTM4B was remarkable. Furthermore, several reports have shown that VEGF could stimulate survivin expression via the PI3K/AKT pathway [22]. This up-regulation of survivin was found to enhance tumor angiogenesis mediated by VEGF [15, 23], suggesting the need for further research into the clinical relevance of these three molecules.

In a further step, strategies should be developed to determine whether the detection of LAPTM4B in combination with other molecules is of diagnostic and prognostic value in assessing breast carcinoma cases. Tang et al. examined LAPTM4B and CD34 proteins in non-small cell lung cancer, and their results revealed that LAPTM4B might promote tumor progression by inducing tumor angiogenesis [24]. Meng et al. indicated that the downregulation of LAPTM4B suppressed tumor migration and invasion and significantly decreased VEGF expression. Subsequently, they verified that the coexpression of LAPTM4B and VEGF resulted in poor prognosis for cervical cancer [17, 18]. Consistent with these findings, our results show that high levels of LAPTM4B and VEGF led to poor clinical outcomes with regard to OS and PFS.

Li et al. revealed that nuclear survivin levels might predict poor survival in breast cancer [25]. The present study indicates that survivin expression is predominantly nuclear rather than cytoplasmic [26], which is line with the majority of previous results. Interestingly, a few reports have indicated that the expression of survivin is nearly equivalent in the nucleus and cytoplasm [27] or only occurs in the cytoplasm [28], which might be attributed to differences in reagents, tissues and clinical stages. In our studies, we separately analyzed the expression of nuclear and cytoplasmic survivin. According to the statistical analyses, nuclear survivin protein expression was dramatically associated with tumor progression and poor survival. Researchers have found LAPTM4B-35 accelerates tumorigenesis in transgenic mice by upregulating the antiapoptotic molecule Bcl-2 and downregulating the proapoptotic molecule Bax. However, the expression of survivin in Ad-AE-infected cells was not altered [16]. Hence, the relationship of the subcellular localization of survivin with other molecules should be further investigated as a greater understanding of this relationship could improve prognostic assessments and individualized therapies.

Fan et al. suggested that LAPTM4B*2 was associated with an increased risk of breast cancer in a cohort of Chinese women [29]. Li et al. demonstrated that MDA-MB-231 cells that had the *2/2 genotype exhibited increased LAPTM4B expression [30]. Similar to these results, our study demonstrated that increased VEGF and nuclear survivin expression occurs in MDA-MB-231 cells.

In conclusion, our findings indicate for the first time that LAPTM4B, VEGF, and survivin protein expression is significantly associated with various clinicopathological characteristics and prognosis in breast cancer patients. In particular, the relation of VEGF and survivin protein levels with the expression of LAPTM4B indicates that they could have clinical potential as promising prognostic markers to identify individuals with poor outcomes and may be regarded as therapeutic targets for breast cancer. However, the limitations in this study included the small sample size and its retrospective nature, such as the limited follow-up time, which could be why the expression of VEGF and survivin was not linked to OS and PFS in the multivariate analysis.

## MATERIALS AND METHODS

### Patients and tissue samples

Specimens were collected from 110 breast cancer patients with stages I-III and 10 patients with benign breast tumors who underwent surgical resection at the Beijing Cancer Hospital between January 2011 and July 2013. All patients provided written informed consent, and none of the patients received chemotherapy, immunotherapy, or radiotherapy before surgery. The Ethics Committee of Beijing Cancer Hospital approved this protocol. All patients with breast cancer were followed-up for the survival analysis until September 2016 (median, 49 months; range, 10–67 months).

### Immunohistochemical staining

Paraffin-embedded samples were cut into four-micrometer sections and stained with hematoxylin and eosin for tumor confirmation. Selected sections were immersed in a retrieval buffer solution for antigen recovery and incubated with a polyclonal rabbit anti-LAPTM4B antibody (dilution 1:200, bs-6542R, Bioss, USA), a polyclonal rabbit anti-VEGF antibody (dilution 1:150, ZA-0509, ZSGB, China) and a monoclonal mouse anti-survivin antibody (dilution 1:2000, produced by our lab) overnight at 4°C. Finally, the slides were stained and mounted. Negative controls were provided by replacing the primary antibodies with normal goat serum.

### Staining evaluation

LAPTM4B, VEGF, and survivin protein expression levels were semi-quantitatively classified. The percentage of positive cells was measured as follows: 0, less than 9% staining; 1, 10% to 25% staining; 2, 26%-50% staining; 3, 51%-75% staining and 4, >75% staining. The staining intensity was evaluated as follows: 0, no staining; 1, weak staining; 2, moderate staining; 3, strong staining. The total score of stained cells was calculated by multiplying the above two scores to define the expression levels: 0, negative expression; 1 to 4, weak expression; 5 to 8, positive expression; 9 to 12, strong expression. Tumor tissues with scores of 0–4 were defined as having low expression and those with scores of 5–12, as having high expression.

### Follow-up

Each patient was scheduled for an examination, which included a physical examination, blood analysis, and computed tomography analysis. Tumor progression was based on clinical, radiological or histological diagnosis, and the site and time of tumor progression were both recorded. Follow-up was performed until September 2016 for 110 patients.

### Cell lines

Breast cancer cell lines (MDA-MB-231, ZR-75-1, T47D, and MCF-7) were kindly provided by Dr. SHOU Cheng-chao from Department of Biochemistry and Molecular Biology, Peking University Cancer Hospital & Institute. MCF-10A cells were obtained from MeiXuan Biological Technology Company Limited of Shanghai, China. They were cultured in appropriate media supplemented with essential materials in the 5% CO_2_ incubator [31].

### Western blot analysis

Protein extracts were separated via 12% SDS polyacrylamide gel electrophoresis and transferred onto PVDF filters. The filters were incubated with a rabbit anti-LAPTM4B polyclonal antibody (dilution 1:1000, AP20870a, ABGENT, China), a rabbit anti-VEGF polyclonal antibody (dilution 1:500, 19003-1-AP, Proteintech, USA) and a rabbit anti-survivin monoclonal antibody (dilution 1:1000, 2808, Cell Signaling Technology, USA) overnight at 4°C. The blots were detected using a chemiluminescence detection system. β-Actin and histone H3 were used as internal controls for the total, cytoplasmic protein expression and nuclear protein expression.

### Statistical analysis

SPSS 18.0 software was used to perform the statistical analyses, and χ2 tests were used to evaluate the associations between the three biomarkers and the clinicopathological characteristics. The follow-up data was analyzed using the Kaplan–Meier method and Cox regression tests. P<0.05 was considered statistically significant.

## Abbreviations

LAPTM4B: lysosome-associated protein transmembrane-4 beta
VEGF: vascular endothelial growth factor
OS: overall survival
PFS: progression-free survival
